# Adenoviruses with an α_v_β integrin targeting moiety in the fiber shaft or the HI-loop increase tumor specificity without compromising antitumor efficacy in magnetic resonance imaging of colorectal cancer metastases

**DOI:** 10.1186/1479-5876-8-80

**Published:** 2010-08-23

**Authors:** Sergio Lavilla-Alonso, Gerd Bauerschmitz, Usama Abo-Ramadan, Juha Halavaara, Sophie Escutenaire, Iulia Diaconu, Turgut Tatlisumak, Anna Kanerva, Akseli Hemminki, Sari Pesonen

**Affiliations:** 1 Cancer Gene Therapy Group, Molecular Cancer Biology Program, Transplantation Laboratory, Haartman Institute and Finnish Institute of Molecular Medicine, University of Helsinki, Finland; 2 HUSLAB, Helsinki University Central Hospital, Finland; 3 Department of Obstetrics and Gynecology, Duesseldorf University Medical Center, Heinrich-Heine University, Germany; 4 Experimental MRI Laboratory, Department of Neurology, Helsinki University Central Hospital, Helsinki, Finland; 5 Department of Radiology, Helsinki University Central Hospital, Helsinki, Finland; 6 Department of Obstetrics and Gynecology, Helsinki University Central Hospital, Finland

## Abstract

**Background:**

Colorectal cancer is often a deadly disease and cannot be cured at metastatic stage. Oncolytic adenoviruses have been considered as a new therapeutic option for treatment of refractory disseminated cancers, including colorectal cancer. The safety data has been excellent but tumor transduction and antitumor efficacy especially in systemic administration needs to be improved.

**Methods:**

Here, the utility of αvβ integrin targeting moiety Arg-Gly-Asp (RGD) in the Lys-Lys-Thr-Lys (KKTK) domain of the fiber shaft or in the HI-loop of adenovirus serotype 5 for increased tumor targeting and antitumor efficacy was evaluated. To this end, novel spleen-to-liver metastatic colorectal cancer mouse model was used and the antitumor efficacy was evaluated with magnetic resonance imaging (MRI).

**Results:**

Both modifications (RGD in the HI-loop or in the fiber shaft) increased gene transfer efficacy in colorectal cancer cell lines and improved tumor-to-normal ratio in systemic administration of the vector.

**Conclusions:**

Antitumor potency was not compromised with RGD modified viruses suggesting increased safety profile and tumor specificity.

## Background

Colorectal cancer is the fourth most common type of cancer in men and the third most common in women worldwide and more than one million people are diagnosed with colorectal cancer every year. Incidence rates have increased during past decades, while 5-year survival rates have improved but remain between 60 to 40% in different countries[1,2]. Metastatic disseminated disease can be cured only rarely and even though early detection and prevention strategies play a key role in improving colorectal cancer statistics, also new therapeutic options are needed. To this end, gene therapy has been of interest to cancer researchers for a few decades and modalities based on adenovirus serotype 5 vectors are one of the most studied strategies. Safety data for adenovirus 5 has been excellent[3-6] and some recent clinical studies have shown some evidence of efficacy for many types of cancer[3-8] including colorectal cancer[9,10]. However, the main disadvantage of the current adenoviral therapies is that the efficacy of tumor transduction limits the efficacy of treatment. In particular, intravenous administration of the vector does not usually allow transduction levels compatible with clinical responses[11,12].

Thus, for successful cancer gene therapy, tumor transduction efficiency needs to be improved, in particular if systemic administration is the goal. Intravenous administration of unmodified adenovirus 5 vectors to mice leads mainly to infection of liver cells. This is mostly due to natural engulfment of adenoviral particles by hepatic macrophages (mainly Kupffer cells)[13] but also several blood factors have been suggested to be involved by bridging the viral capsid proteins to heparan sulphate proteoglycans (HSPG) and some other receptor molecules on the surface of hepatocytes[14-20]. Therefore several attempts have been made to detarget the liver for more appealing systemic bioavailability. Coxsackie- and adenovirus receptor (CAR) binding ablation by changing amino acid residues of the fiber binding motif has been suggested to avoid vector ending into the liver hepatocytes but this modification has been shown to be an inadequate to change the biodistribution of the virus [21]. Cell surface integrins are also important players in the adenovirus serotype 5 entry. After binding to CAR, adenoviral penton base Arg-Gly-Asp (RGD) motif interacts with cellular αvβ integrins to facilitate internalization[22,23]. However, even double ablation of CAR and integrins fail to reduce Ad5 hepatocyte tropism in systemic delivery[21,24-27]. In the absence of CAR, Lys-Lys-Thr-Lys (KKTK) domain in the fiber shaft has been suggested to play a major role in viral internalization via low affinity binding with HSPG[27-29] and a mutation in this domain has been shown to decrease viral tropism towards hepatocytes[27,29].

It has been shown earlier with replication deficient viruses that in comparison with unmodified virus, increased tumor cell transduction is achieved with adenoviruses with RGD moieties in the HI loop of the fiber or in the KKTK domain of the fiber[30]. Furthermore, mutation of the KKTK domain ablated binding to HSPGs and led to reduced liver cell transduction and improved tumor-to-liver transduction ratio[30]. We hypothesize here that the antitumor efficacy of systemically administered replicating competent adenovirus can be increased by targeting virus towards cell surface αvβ integrins and by simultaneously abrogating liver transduction with mutated KKTK domain of the fiber shaft. To this end, novel spleen-to-liver metastatic colorectal cancer mouse model was used and the antitumor efficacy was evaluated with magnetic resonance imaging (MRI).

## Methods

### Cell lines

All human colorectal cancer cell lines were acquired from ATCC (American Type Culture Collection), cultured in the recommended growth media with 10% fetal calf serum (FCS) and maintained in a humidified atmosphere at 37°C and 5% CO2.

### Viruses

Non-replicating viruses were produced by substitution of the E1 region for a marker gene cassette. All non-replicating viruses contain a green fluorescent protein (GFP) and a firefly luciferase (Luc) expression cassette under the constitutive cytomegalovirus promoter replacing E1. For all non-replicating viruses, cloning and large-scale production has been described before (see Table 1 for references). Replication competent viruses WT-RGD and WT-RGDK were kindly provided by Professor Ramon Alemany (Translational Research Laboratory, Institut d'Investigació Biomèdica de Bellvitge (IDIBELL)-Institut Català d'Oncologia, L'Hospitalet de Llobregat, Barcelona, Spain). A summary of all viruses is given in Table 1.

**Table 1:** Description of viruses used in the study

Virus		Capsid modification	References
**Replication deficient viruses**^**a**^	AdTL	Wild type 5 capsid	
	
	DATL	-Y477A substitution in DE loop of fiber knob for CAR ablation	
		-Penton base's RGD domain mutated to RGE for α_v_β integrin ablation	[41]
		-6xhistidine carboxy-terminal tag for the propagation in 293.HissFv.rec cells	
	
	AdTLG	-Fiber shaft's KKTK domain mutated to GATK for HSPG ablation	[27]
	
	AdTLGR	-RGD insertion in HI loop of fiber knob for α_v_β integrin targeting	
		-Fiber shaft's KKTK domain mutated to GATK for HSPG ablation	[27]
	
	AdTLYG	-Y477A substitution in DE loop of fiber knob for CAR ablation	
		-Fiber shaft's KKTK domain mutated to GATK for HSPG ablation	[21,27]
	
	AdTLYGR	-Y477A substitution in DE loop of fiber knob for CAR ablation	
		-RGD insertion in HI loop of fiber knob for α_v_β integrin targeting	
		-Fiber shaft's KKTK domain mutated to GATK for HSPG ablation	[21,27]
	
	AdTLY	-Y477A substitution in DE loop of fiber knob for CAR ablation	[21]
	
	Ad5luc1RGD	-RGD insertion in HI loop of fiber knob for α_v_β integrin targeting	[42]
	
	AdTLRGDK	-Fiber shaft's KKTK domain mutated to RGDK for αvβ integrin targeting	[30]
		-HSPG ablation via mutated KKTK	

**Replicating viruses**	WT	-Replicating wild type 5 virus	
	
	WT-RGD	-RGD insertion in HI loop of fiber knob for α_v_β integrin targeting	[43]
	
	WT-RGDK	-Fiber shaft's KKTK domain mutated to RGDK for α_v_β integrin targeting	[30]
		-HSPG ablation via mutated KKTK	

^a^All replication deficient viruses are deleted for E1A and have both *luciferase *(*Luc*) and *green fluorescent protein *(*GFP*) as marker genes.

CAR, coxsackie virus and adenovirus receptor; HSPG, heparan sulphate proteoglycan; VP, viral particles; pfu, plaque forming unit.

### Animals

All animal experiments were conducted according to the rules set by the Provincial Government of Southern Finland (permit number ESLH-2008-01986/Ym-23). Pathogen-free, 3-4-week-old female NMRI nude mice were purchased from Taconic (Ejby, Denmark) and quarantined for 2 weeks. The animals were fed ad libitum and maintained in a HEPA-filtered environment with cages, food, and bedding sterilized by autoclaving.

### Analysis of the transgene expression

Cells were infected with replication deficient, luciferase-expressing viruses at 1000 viral particles per cell (VP/cell) in 200 μl of 2% FCS for 30 min, and then washed and incubated with complete growth medium at 37°C. After 24 h, luciferase assay (Luciferase Assay System, Promega, Madison, WI, USA) was performed according to the manufacturer's instructions.

### Viral oncolytic potency in human colorectal cancer cells

Cells were infected with replication competent viruses or non-replicating control virus, and after 1 h, infection medium was replaced with medium containing 5% FCS, which was changed thereafter every other day. 8 to 11 days later (at the optimal time point for each cell line), cell viability was analyzed with the mitochondrial activity-based 3-(4,5-dimethylthiazol-2-yl)-5-(3-carboxy-methoxyphenyl) -2-(4-sulfophenyl)-2H- tetrazolium (MTS) assay (Cell Titer 96 AQueous One Solution Cell Proliferation Assay; Promega, Stockholm, Sweden).

### Spleen-to-liver tumor model

The surgical procedure was similar to what has been previously described[31]. Briefly, mice were anesthetized with ketamine (Ketaminol^® ^75 mg/kg; Intervet, Boxmeer, Netherlands)/dexmedetomidine (Dexdormitor^® ^1 mg/kg; Orion Pharm, Espoo, Finland) admixture and the spleen was exteriorized through a left lateral flank incision. Tumors were established by intrasplenic injection of 2 × 10e6 HCT116 cells suspended in 50 μl of serum-free growth media using a 27-gauge needle. The injection site of the spleen was pressed with a cotton stick wet in iodine-polividone solution (Betadine^®^; Leiras, Helsinki, Finland) in order to remove extravasated cells and ensure hemostasis. The peritoneum and skin were closed in a single layer using surgical thread. Finally, atipamezole (Antisedan^® ^1 mg/kg; Orion Pharm, Espoo, Finland) was injected subcutaneously to reverse anesthesia.

### Biodistribution study

21 days after intrasplenic injection of HCT116 cells, 3 × 10e10 VP of AdTL, AdTLGR, or AdTLRGDK in 150 μl of PBS were injected through the tail vein of NMRI nude mice. After 48 hours, mice (n = 5 in each group) were sacrificed and organs and tumors were harvested for luciferase analysis. To separate between tumors and organs, tumor tissue and normal liver/spleen tissues were microdissected by visual inspection. Data was normalized for protein concentration by Pierce BCA Protein Assay Kit^® ^(Thermo Scientific, Rockford, IL, USA).

### Antitumor efficacy *in vivo*

Tumors were implanted as described above. On days 23 and 24 after cell injection, mice were treated with two intravenous injections of 3 × 10e10 VP of WT, WT-RGD, or WT-RGDK in 100 μl volume of PBS (n = 4, 11, and 9, respectively). Mock animals (n = 9) were treated with PBS only. Tumor volume was followed up by MRI of the abdomen. Mice were imaged under isoflurane (Baxter, Helsinki, Finland) anesthesia. 30 minutes before imaging, 1 mg/kg of contrast agent Endorem (Guerbet, Roissy CdG Cedex, France) in 100 μl volume was administered intravenously.

MRI studies were performed with a 4.7 T scanner (PharmaScan, Bruker BioSpin, Ettlingen, Germany) using a 90-mm shielded gradient capable of producing a maximum gradient amplitude of 300 mT/m with an 80-μs rise time. A linear birdcage RF coil with an inner diameter of 38 mm was used. T2-weighted images were acquired using rapid acquisition with relaxation enhancement (RARE) sequence (TR/TE_eff _= 3767/36 ms, matrix size = 256 × 256, Rare Factor = 8, field-of-view = 33 × 33 mm^2^, 32 slices, slice thickness = 0.7 mm, number of averages = 8).

Tumor tissue areas in the liver were measured in every slice and a total tumor volume was calculated using the formula: ∑ (Area*slice height) or ∑ (Area*0.7). In order to distinguish hepatic tumor tissue from vessels or other structures present in the liver, all images were compared to a baseline image of each mouse taken before tumor implantation. Daily volumes of hepatic tumor tissue were normalized to tumor volume one day before treatment. The survival of animals was followed.

### Viral replication in tumor tissue

29 days after intrasplenic injection of tumor cells, mice were treated with 3 × 10e10 VP of WT, WT-RGD, or WT-RGDK in 100 μl of PBS, or PBS alone (mock) (n = 5 in all groups, except for WT-RGDK n = 6). 3 days after treatment, mice were sacrificed and hepatic tumors were harvested, homogenized and diluted in growth media. After three freeze and thaw cycles (-80C/room temperature), tumor lysates were centrifuged, supernatant was collected and added to 293 cells to perform TCID50 test. Plaque forming units per ml (pfu/ml) values were normalized for total hepatic tumor volume and the final result was given as amount of pfu/tumor.

### Statistics

All analyses were done with SPSS 15.0 for Windows. One-way analysis of variance (ANOVA) followed by Dunnett's Pairwise Multiple Comparison t-test was used to analyze the differences in the cell killing potency of viruses *in vitro *and tumor growth and virus replication *in vivo*. Mann-Whitney test was used to analyze the differences in the biodistribution and tumor-to-organ ratios. Survival data was plotted into a Kaplan-Meier curve and groups were compared pair-wise with log-rank test. A value for p < 0.05 was considered statistically significant.

## Results

### Gene transfer to human colorectal cancer cells

**Figure 1:**
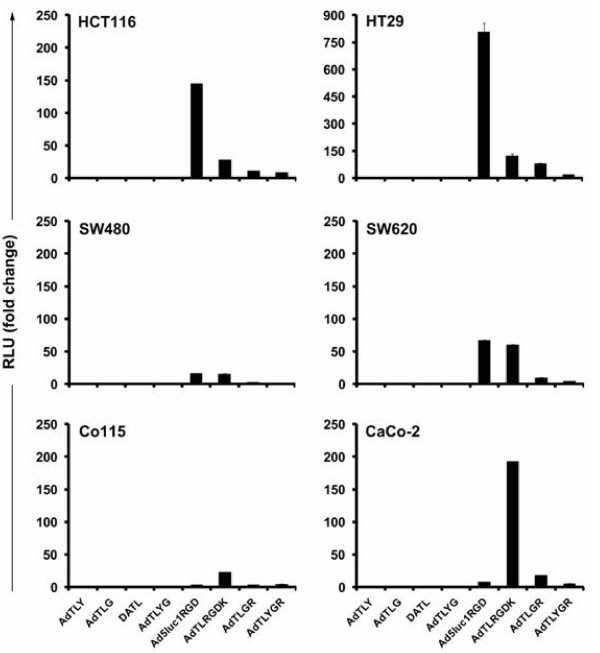
**Gene transfer to human colorectal cancer cells**. Adenoviral vectors targeted for α_v_β integrins via Arg-Gly-Asp (RGD) modification in the HI loop (Ad5luc1RGD) or the shaft domain (AdTLRGDK) of the fiber showed enhanced gene transfer to human colorectal cancer cell lines. Cells were infected with 1000 VP/cell and luciferase activity was measured 24 hours later. Data is presented as relative light units (RLU) normalized for gene expression of Ad5 control virus AdTL. Each data point represents the mean of three replicates ± SEM.

Six established colorectal cancer cell lines were infected with a panel of capsid modified viruses and control virus with an unmodified Ad5 capsid (Figure 1). A Y447A substitution was engineered into the DE loop of the fiber knob for CAR binding ablation (AdTLY). This decreased transgene expression in comparison with Ad5 in all six cell lines confirming the crucial role of CAR in vitro infection in colorectal cancer cells. Also ablation of binding to HSPG (AdTLG) reduced gene transfer compared to Ad5. As expected, double ablations for CAR and αvβ integrin (DATL) or CAR and HSPG (AdTLYG) binding reduced gene expression levels as well. Since CAR/HSPG ablation affects significantly the ability of viruses to infect 293 cells, the usual assessment of pfu titers cannot be performed. Therefore, a direct comparison of VP to pfu ratios between viruses cannot be done and it is possible that some of the differences observed between the groups are due to variable viability of viral preparations.

Targeting viruses to cell surface αvβ integrins by inserting RGD tripeptide motif into the HI loop of the fiber knob (Ad5lucRGD) or into the fiber shaft KKTK domain (AdTLRGDK) increased the expression of transgene in all tested cell lines in comparison with AdTL. Interestingly, the optimal location for the RGD modification in the fiber varied between cell lines. In HCT116 and HT29 cells, RGD in the HI loop of the fiber was the most potent and increased luciferace expression 145 and 804 -fold in comparison with the wild type virus, respectively. In Co115 and CaCo-2 cells, the highest gene expression levels were displayed by the virus with the RGD in the HSPG binding ablated fiber shaft (22 and 192 fold increase, respectively). For two cell lines (SW480 and SW620), both RGD variants were equally effective. The RGD mediated enhancement in transgene expression was partially abolished by introducing additional modification(s) in the fiber to ablate binding either from HSPG (AdTLGR) or from both HSPG and CAR (AdTLYGR). In five out of six cell lines, RGD modification in the HI loop increased transduction efficiency in comparison with control virus even if the vector interaction with CAR and HSPGs was abrogated (AdTLYGR).

### Biodistribution of adenoviral vectors with RGD modification in the capsid

**Figure 2:**
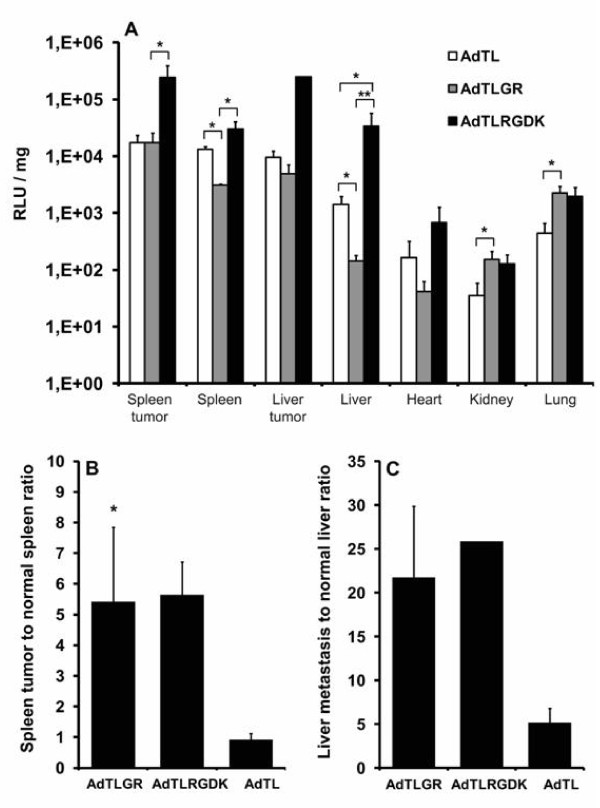
**Biodistribution of adenoviral vectors with RGD modification in the capsid**. Mice bearing intrasplenic and intrahepatic tumors were injected via tail vein with 3 × 10e10 VP and organs/tumors were harvested two days later. The number of 5 animals was treated in each group. (A) Luciferase expression of organs was analyzed. Data are presented as relative light units (RLU) after normalization for protein concentration. Each bar represents mean ± SEM. (B) Spleen tumor to normal spleen ratio of transgene expression. (C) Liver tumor to normal liver ratio of transgene expression. *, p < 0.05; **, p < 0.01.

Since αvβ integrin targeted vectors showed an increased transduction efficacy in colorectal cancer cells in vitro, the biodistribution of RGD modified viruses in vivo was tested in metastatic colorectal cancer spleen-to-liver model. In addition to tumor targeting RGD moieties, viruses had also a mutated KKTK domain of the fiber shaft, which has been shown earlier to decrease viral tropism towards hepatocytes[27]. NMRI nude mice bearing intrasplenic and intrahepatic HCT116 tumors were systemically injected with 3 × 10e10 VP of AdTL (Ad5 control), AdTLGR (RGD in the HI loop; KKTK mutated to GATK), or AdTLRGDK (KKTK mutated to RGDK) (Figure 2A). At 48 hours, luciferase activity and protein concentration of organs and tumors (primary spleen tumors and metastatic liver tumors) were measured. The best tumor transduction was achieved with AdTLRGDK, which displayed the highest transgene expression in both spleen tumors and liver metastases. For spleen tumors, transgene expression of AdTLRGDK was significantly higher in comparison with AdTLGR virus (p = 0.047) and the similar trend was seen in comparison with the Ad5 control. In the liver tumors, no statistically significant differences were seen between viruses due to low number of tumors in each treatment group (n = 2, 2, and 1 for AdTL, AdTLGR, and AdTLRGDK, respectively). Both RGD modified viruses showed an increased tumor-to-normal ratio in transgene expression (Figures 2A and 2B). Virus with RGD modification in the HI loop (AdTLGR) increased tumor cell transduction in the spleen and liver tumors 6 (p = 0.025) and 4 fold in comparison with unmodified virus, respectively. Similarly, virus with RGD modification in the KKTK domain of the fiber shaft (AdTLRGDK) increased spleen and liver tumor transduction 6 and 5 fold, respectively.

Interestingly, AdTLRGDK and AdTLGR viruses showed significant differences in their biodistribution. In the normal liver tissue, AdTLGR displayed significantly lower transgene expression if compared to AdTL (p = 0.047), whereas AdTLRGDK showed significantly higher expression in comparison with AdTL (p = 0.047). A similar trend was seen in the spleen, where AdTLGR demonstrated lower gene transfer in comparison with AdTL (p = 0.014), but the difference between AdTLRGDK and AdTL was not significant (p = 0.14). For kidneys and lungs, the only statistically significant difference was enhanced gene transfer of AdTLGR in comparison with AdTL (p-values of 0.047 and 0.027, respectively). In the heart, no significant differences in the efficacy of gene transfer were seen between viruses.

### Cell killing potency of RGD modified viruses in vitro

**Figure 3:**
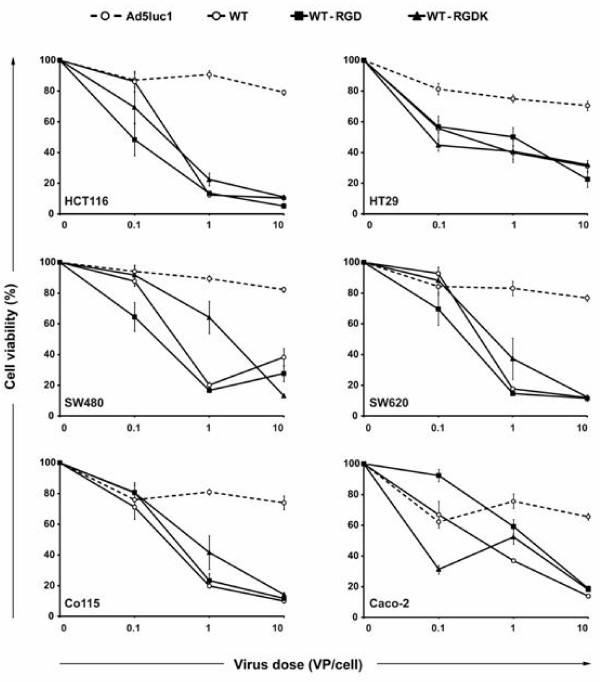
**Cell killing potency of RGD modified viruses in vitro**. Viruses with RGD modification in the capsid display effective killing of colorectal cancer cell lines. Cells were infected with replication competent (WT-RGD, WT-RGDK, WT) or non-replicating (Ad5luc1) viruses and the cell killing potency was assessed with the MTS assay. Data are presented as relative cell viability normalized to mock (growth medium) infected cells. Each data point represents the mean of six replicates ± SEM.

Oncolytic potency of replication competent viruses WT-RGD, WT-RGDK, and control virus WT was analyzed in six colorectal cancer cell lines in vitro by MTS assay (Figure 3). At the lowest viral dose (0.1 VP/cell), RGD modified viruses killed cells more effectively in comparison with WT in three out of six cell lines. At higher viral doses, however, RGD insertion in the HI loop of the fiber (WT-RGD) or in the shaft domain (WT-RGDK) did not increase the oncolytic potency and all three replication competent viruses showed an equal cell killing potency in all six established colorectal cancer cell lines. The E1-deleted Ad5 control virus did not cause oncolytic cell death in any of the cell lines.

### Antitumor efficacy of RGD modified viruses in the spleen-to-liver colorectal cancer model

**Figure 4:**
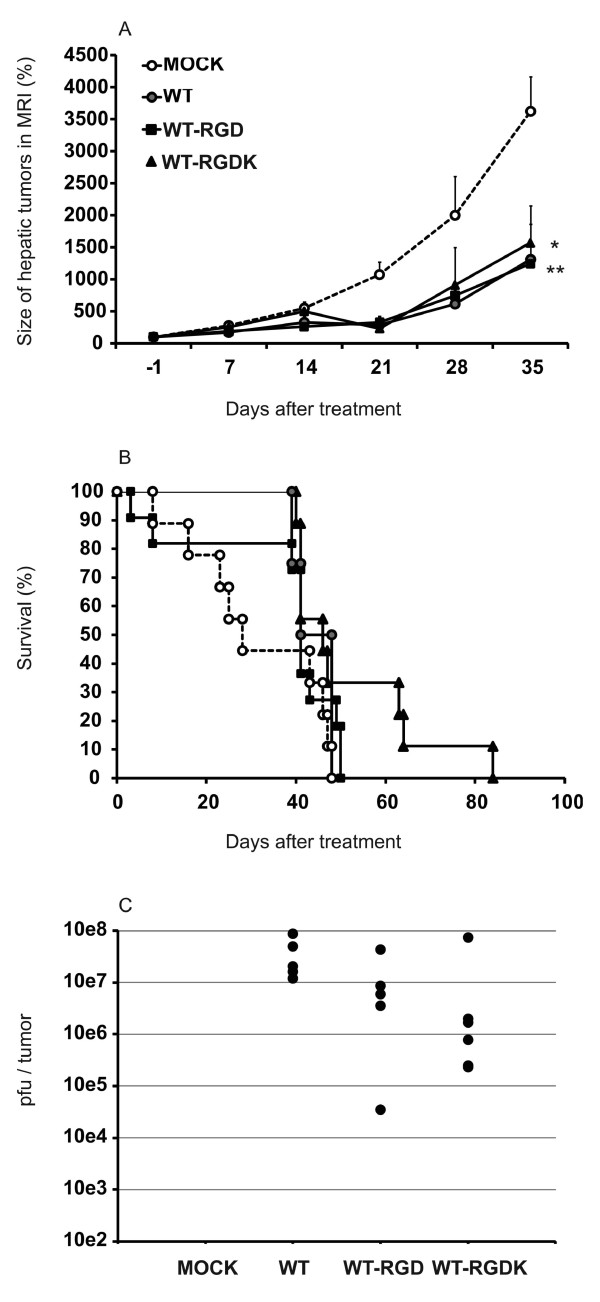
**Antitumor efficacy of RGD modified viruses in the spleen-to-liver colorectal cancer model**. Enhanced therapeutic effect of RGD modified replication competent adenoviruses in spleen-to-liver colorectal cancer model. To imitate clinical metastatic colorectal cancer, hepatic tumors were induced in mice by intrasplenic injection of HCT116 colorectal cancer cells. WT, WT-RGD, or WT-RGDK viruses at dose of 3 × 10e10 VP were injected via tail vein in two consecutive days (days 23 and 24). (A) Hepatic tumor growth was followed with MRI thereafter. Relative tumor volumes normalized to the day before virus treatment (day -1) tumor volumes are presented. Each data point represents mean of 2 to 11 measurements ± SEM. *, p < 0.05; **, p < 0.01. (B) The survival of animals was assessed. No statistically significant differences in the survival of animals between treatment groups were observed. (C) Virus replication in liver tumors was assessed three days after systemic administration. Mock animals received PBS only. Pfu/ml values obtained from TCID50 test were normalized for tumor volume. Each dot represents an individual liver tumor. All viruses replicated in the liver tumor tissue and no statistically significant differences were seen between virus treated groups.

**Figure 5:**
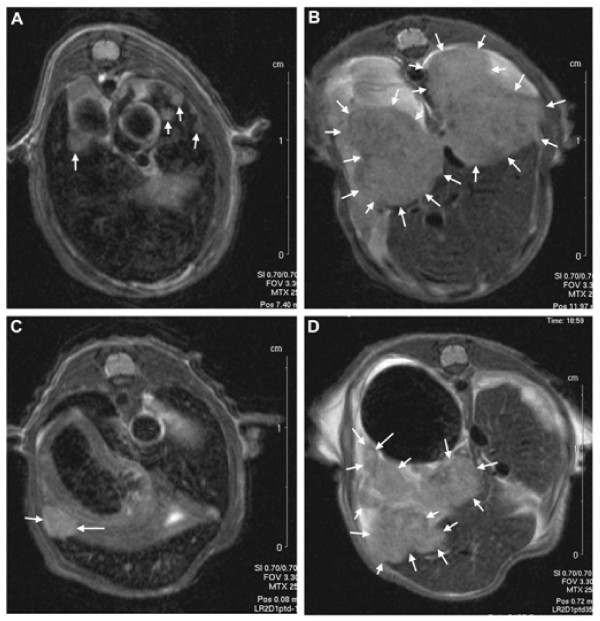
**Viral replication in the liver tumors**. The growth of liver metastasis was analyzed with magnetic resonance imaging (MRI). Tumors are marked with arrows. Picture of liver metastasis of mock treated (PBS) animal (A) 1 day before treatment and (B) on day 35 after treatment. (C) Picture of liver metastasis of WT-RGD treated animal one day before treatment and (D) on day 35 after WT-RGD treatment.

Colorectal cancer cells (HCT116) were injected into the spleen of NMRI nude mice and intrasplenic and hepatic tumors were allowed to grow for 23 days. Two intravenous injections of viruses were given on consecutive days, and hepatic tumor volumes were followed by MRI thereafter (Figure 4A). By day 21, the growth rate of hepatic tumors was inhibited in all virus treated groups if compared to mock treated animals. At the end of the experiment on day 35, only WT-RGD (p = 0.004) and WT-RGDK (p = 0.026) treated animals showed statistically significant reduction in tumor growth in comparison with mock animals, while borderline significance (p = 0.054) was observed between WT and mock groups. Treatment with WT, WT-RGD and WT-RGDK led to median survival of 44.5, 41, and 46 days, respectively, while median survival for mock treated animals was 28 days (Figure 4B). In comparison with mock, none of the treatments improved survival statistically significantly. However, three of the mice treated with WT-RGDK virus survived 15, 16, and 36 days longer than the last mouse in the mock group (p = 0.055 between mock and WT-RGDK). Typical results of MRI are presented in Figure 5.

### Viral replication in the liver tumors

Hepatic tumors induced by intrasplenic inoculation of the HCT116 cells were harvested three days after intravenous virus administration to assess the amount of actively replicating virus in the tumors by TCID50 method (Figure 4C). All tumors from virus treated animals had measurable titers for replicating virus whereas no virus replication was detected in tumors of PBS treated mice. However, no statistically significant differences in the functional titers were observed between different viruses and active virus was found in all tumors collected from virus treated animals.

## Discussion

Numerous papers suggest that other entry mechanisms in addition to CAR binding are important in mediating adenovirus serotype 5 distribution in vivo[21,32]. Here, we tested the biodistribution of αvβ integrin targeted Ad5 vectors able or unable to bind to HSPG. In line with an earlier study by Bayo-Puxan et al[27], a virus with RGD modification in the HI loop and mutation of the fiber shaft KKTK domain to GATK (the HSPG binding ablation) showed reduced liver and spleen transduction in comparison with wild type virus. This demonstrates the potency of mutated KKTK to GATK in the fiber shaft to detarget the liver in vivo. We used different tumor cell lines and tumor models than what had been used in previous reports, suggesting that the phenomenon is not a cell line or tumor model specific finding.

It has been suggested earlier, that GATK mutation in the KKTK domain (AdTLGR) may reduce the potency of tumor targeting by the RGD modification in the HI-loop[27]. However, in contrast to earlier findings showing a decreased tumor cell transduction in subcutaneous A549 xenografts[27], no reduction in liver and spleen tumors transduction was seen with AdTLGR virus in comparison with unmodified virus. In the contrary, a significantly increased tumor to normal spleen gene delivery ratio was seen with AdTLGR. This suggests that RGD modification in the HI loop of KKTK mutated virus might be useful to increase tumor specificity. However, in our experiments the efficacy of this modification varied between cancer cell types and tumor models used. HCT116 cells are typical representatives of clinical colorectal cancers[33-35] in that they express high levels of av integrins[36] which might partially explain the good transductional targeting achieved with RGD modified viruses in this study.

KKTK mutation to RGDK might also theoretically detarget vector from the liver and this has been tested earlier in C57BL/6 mice[30]. As a result, marginal decrease in the liver transduction was seen accompanied by an increase in the tumor cell transduction[30]. In our model, KKTK domain mutation to RGDK significantly increased transgene expression in the liver in comparison with unmodified virus, and similar trend was seen in all the other organs as well. This may have been caused by the opposite effects of HSPG ablation and RGD insertion; while the former ablates transduction via HSPG, the latter increases delivery through av integrins. However, since tumor cell transduction was increased more than transduction to normal tissue, increasing trend in tumor-to-organ ratio was seen in comparison with unmodified virus.

Overall, replacing KKTK with RGD in the fiber shaft emerged as the optimal fiber mutation. As the most relevant control for efficacy experiments, we selected an established RGD modification of the capsid (KKTK intact, RGD in HI loop), as this virus has already been safely used in a clinical trial[37]. In vitro, antitumor efficacy was increased with both RGD modified viruses in comparison with unmodified virus in 3 out of 6 cell lines. However, as expected in vitro conditions, where most viruses are expected to eventually enter cells as they have no other place to go to, differences were small.

In an advanced orthotopic model of metastatic colorectal cancer, tumor growth was significantly reduced by RGD modified viruses in comparison with untreated animals. In contrast, the difference between untreated animals and animals treated with wild type control virus was not significant. Overall, RGD modification in the HI-loop or in the KKTK domain of the shaft might be useful to increase an antitumor efficacy of an oncolytic adenovirus. However, additional targeting strategies are needed (e.g. transcriptional targeting) to increase tumor specificity of these viruses before testing these constructs in humans.

In this study, the feasibility of using MRI analysis for following tumor growth was evaluated. From an ethical point of view, this method reduces the number of mice needed in each group since individual tumors inside body cavity can be followed. MRI allows also the use of non-subcutaneous tumor models for tumor growth follow-up. Tumors grown in the correct organ likely resemble the human disease more closely than subcutaneous tumor models[38,39]. Therefore, in vivo MRI analysis for the tumor growth follow-up may emerge as a valuable tool for future studies.

Targeting adenovirus towards av integrins is an effective way to increase tumor cell transduction in vitro, as was shown by an increased transduction of colorectal cancer cells with RGD targeted vectors, even if the vector interaction with CAR and HSPGs was abrogated. However, in vivo the situation is more complicated. Several studies have shown that adenovirus vector targeting in vivo is not mediated only by vector binding properties to cell surface receptors and vector biodistribution does not correlate with in vitro data. This suggests that many factors, including anatomical barriers[40], vascular access or blood factors[14-17] play a role in determining the faith of systemically administered adenoviral vectors in vivo. Also the use of different animal and tumor models makes the interpretation and comparison of results complicated and it is not well understood how these models correlate with humans. Furthermore, most of the existing data are based on immune deficient mouse models and whether it can be applied in humans where the immune system makes the life of an adenovirus much tougher, requires further study.

RGD modification in the KKTK domain of the fiber shaft may have potential to increase the overall antitumor efficacy of the oncolytic adenovirus. However, transductional targeting may not be enough to make the virus usable in humans and therefore additional targeting strategies have been utilized. For instance, transcriptional targeting of the virus via tumor specific promoters or with mutations which are transcomplemented by mutations in tumor cells (e.g. 24 bp deletion in E1A; "D24") would make the virus more tumor specific and increase efficacy and safety.

## Conclusions

Here, the antitumor potency of RGD modified viruses was proved to be equal, or marginally increased, in comparison with unmodified wildtype 5 virus. In addition, tumor targeting was improved significantly. These results suggest that RGD modification increases the specificity and safety of oncolytic adenovirus without compromising the efficacy in an experimental model and gives rationale for testing the RGD modification in the context of oncolytic adenoviruses in humans.

## Competing interests

The authors declare that they have no competing interests.

## Authors' contributions

The work presented here was carried out in collaboration between all authors. SLA, GB, SP and AH defined the research theme and designed methods and experiments. Laboratory experiments were carried out by SLA with assistance of GB, ID and SE. Animal work was carried out by SLA with the assistance of SE. The mouse model was designed and developed by SLA. MRI methods were validated by UAR and SLA, interpretation of MR images was done by JH and SLA and quantification of tumor volumes and subsequent analysis of the data by SLA. Statistical calculations were performed by SP. SLA, TT and SP analyzed the data, interpreted the results and wrote the paper. All authors have contributed to, seen and approved the manuscript.
